# Evolving strategies for virus discovery

**DOI:** 10.1099/mgen.0.001785

**Published:** 2026-07-08

**Authors:** Amanda Araujo Serrao de Andrade, Andrea Silverj, Theodore Josephs, Ann C. Gregory

**Affiliations:** 1 Department of Biological Sciences, University of Calgary, Calgary, AB, T2N 1N4, Canada; 2 Snyder Institute for Chronic Diseases, Cumming School of Medicine, University of Calgary, Calgary, AB, T2N 1N4, Canada

**Keywords:** metagenomics, phage, viral ecology, virome, virus

## Abstract

Viruses interact with all domains of life and play fundamental roles in shaping biological systems from individual hosts to global ecosystems. Yet their identification remains difficult due to a lack of a universal marker gene and the extensive diversity of viral genomes. Despite this, the speed of viral discovery is quickly increasing, driven by the growing number of virome studies, improved sequencing technologies and the decreased cost of sequencing. In this review, we examine the evolution of virus identification approaches from classical and molecular methods to contemporary genome-resolved and computational frameworks. By aggregating genome-resolved virome studies from 2010 to early 2026 that meet defined criteria (*n*=502), we synthesize the current landscape of virus identification methods, including similarity-based, sequence-based artificial intelligence (AI) and hybrid approaches. We also highlight the key limitations of the current methods, particularly biases in reference databases that contribute to persistent viral ‘dark matter’. Finally, we identify emerging opportunities for the field in structure-based and AI-driven approaches that extend detection beyond sequence similarity and outline how these integrative frameworks are poised to improve virus discovery across ecosystems.

**Impact Statement**

Over the past 5 years, sequence-based artificial intelligence protein structure prediction has transformed the landscape of biology, opening access to an unprecedented diversity of protein folds. In viromics, we believe this will revolutionize how viruses are identified and classified, as viral protein structures make it possible to model distant homologous relationships that are only detectable at this level. We highlight the potential of these new methods compared to currently available approaches, for which we provide a comprehensive overview by listing and summarizing all major studies in the field from 2010 to the present. For most of the modern viromics era, analyses have relied solely on sequence data, limiting the detection of highly divergent viruses in metagenomic datasets. We anticipate that this emerging framework based on protein structure comparisons will reshape how viruses are identified and classified across ecosystems and provide a path towards a more complete view of the virosphere.

## Data Availability

The scripts used to generate the plots and tables presented in the manuscript are available at https://github.com/IntegrativeViromicsLab/micro_gen_review.

## Introduction

Viruses have historically been among the most difficult biological entities to identify and classify. While bacteria were first observed in 1676, viruses remained invisible to science for nearly two more centuries [1,3]. This delay reflects not only technological but also conceptual challenges, as viruses are too small to be visualized via light microscopy and they lack shared cellular features that underpin classical definitions of life. Consequently, the development of virus identification approaches has long lagged behind those used for bacteria and archaea.

Beyond their small size, one of the most difficult obstacles to virus discovery is the absence of a universal marker gene. In bacteria and archaea, the 16S rRNA gene provides a conserved genetic marker that enables broad detection, classification and comparative analysis across ecosystems [4]. No equivalent marker exists for viruses. Viral genomes comprise DNA or RNA, single- and double-stranded forms, segmented and non-segmented architectures and highly diverse gene repertoires arising from higher mutation rates, recombination and horizontal gene transfer [5,6]. This lack of universal conservation has made marker-based virus identification inherently biassed towards previously characterized lineages.

As a result, virus identification has progressed through a succession of different methodological frameworks, each shaped by the available technology and prior knowledge. Early experimental approaches inferred the presence of viruses indirectly, relying on observable effects such as disease transmission, host cell lysis or the passage of infectious agents through filters that excluded bacteria, rather than direct visualization of the viral particles [3,7, 8]. Later molecular methods focused on conserved genes within specific viral groups to support detection and classification [9,11]. More recently, high-throughput sequencing and metagenomics have transformed virus discovery by enabling the detection of large numbers of uncultivated viruses directly from the environment and host-associated samples [12,13]. Despite these advances, genomic-centric approaches remain limited by reference database coverage, assumptions of sequence similarity and the persistence of ‘viral dark matter’ [14,15].

In this review, we trace the evolution of virus identification methods from pre-genomic foundations to contemporary homology-based, artificial intelligence (AI)-driven and structure-informed approaches. By integrating historical context with current practices and emerging directions, we examine both the enduring challenges posed by viral diversity and the opportunities created by advances in computation, protein structure prediction and large-scale data integration.

## Foundational approaches to virus identification

**Fig. 1.**
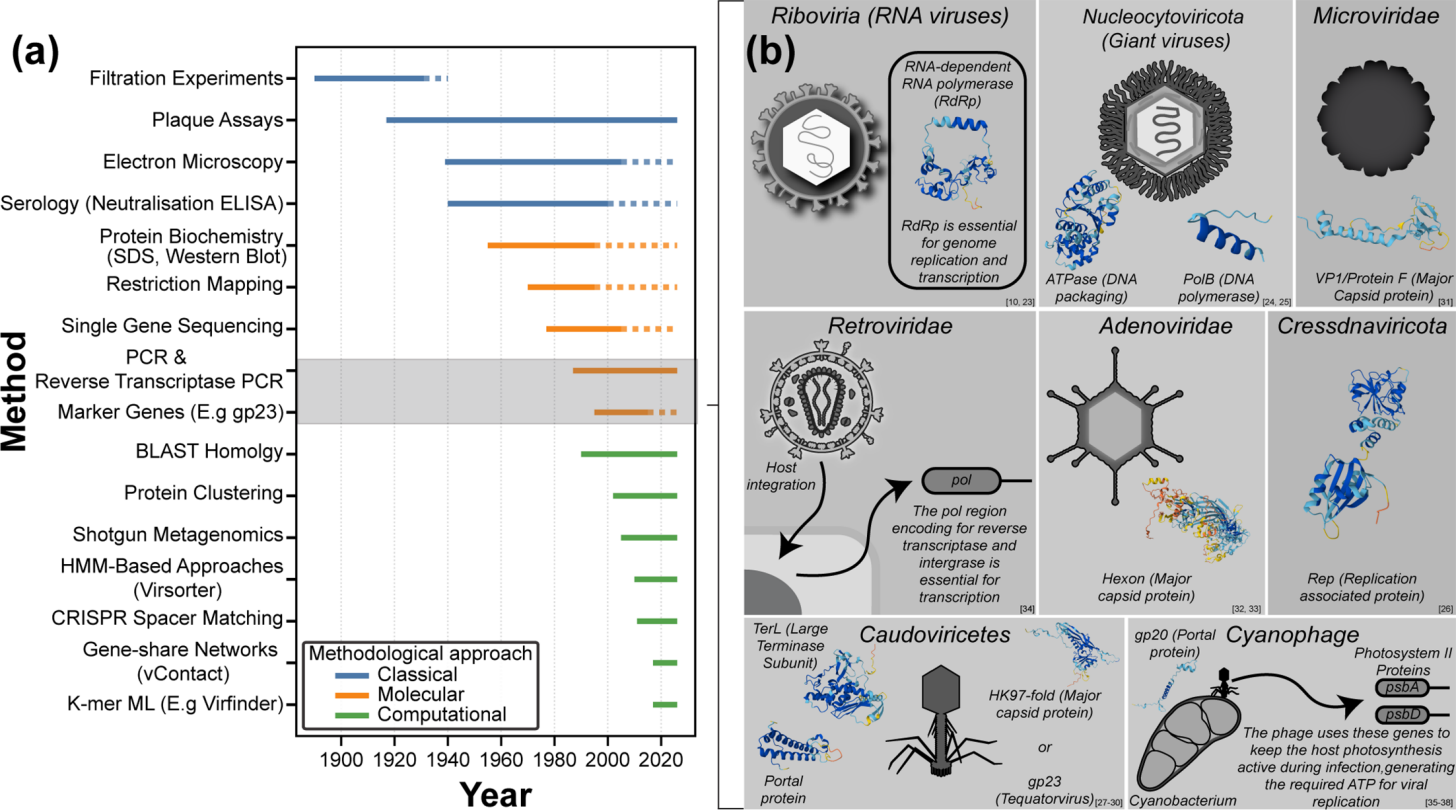
Methodological advances and core marker genes in viral classification and phylogeny. (**a**) Timeline of major viral identification methodologies (1885–present). Methods are grouped by approach: classical (phenotypic assessment and visualization), molecular (sequence-based) and computational (genome-scale and AI). Solid bars represent periods when a methodology was the primary standard for viral discovery. Dash lines indicate reduced reliance, marking the historical point where newer technologies emerged and older methods transitioned to supplementary tools. (**b**) Core marker genes for viral classification and identification. Representations of major viral groups, including RNA viruses (*Riboviria*), ssRNA (*Retroviridae*), dsDNA (*Adenoviridae*, *Caudoviricetes*, *Nucleocytoviricota* and cyanophage) and ssDNA viruses (*Microviridae* and *Cressdnaviricota*). Highlighted are key structural proteins (e.g. Hexon and HK97-fold) and replication enzymes (e.g. RdRp, PolB and Rep). The cyanophage panel (bottom right) illustrates the viral hijacking of host metabolism (photosystem II: psbA and psbD) to support viral replication. Bracketed numbers indicate citations. Protein structures were sourced from AlphaFold [118,120, 155] (accessions: A0A481YZH0, A0A286Q6J9, A7IY91, A0A2U7NLV3, C6K7K0, P85987, Q71F16 and X2J3M8 [125]; accessions: Q91EK3 and P11819 [126]).

Before the advent of genomics, virus identification relied on indirect experimental evidence rather than direct observation of viral particles. In the late nineteenth century, Adolph Mayer’s work on tobacco mosaic disease showed that infectious material could pass through bacteria-retaining filters, indicating the existence of a different class of infectious agents distinct from bacteria [16,17]. Building upon Adolph Mayer’s work, Dmitri Ivanovsky (1892) and Martinus Beijerinck (1898) independently showed that filtered plant extracts remained infectious, establishing filterability as a defining property of viruses [2,3, 18]. While these early classical experiments could not visually confirm the presence of viral particles, they did initiate a methodological cascade that has continued to expand our toolkit for understanding viral diversity, many of which are still used today (Fig. 1a).

The discovery of bacteriophages by Frederick Twort (1915) and Félix d’Hérelle (1917) introduced plaque assays as a quantitative and observational method for detecting viruses through zones of host lysis on bacterial lawns [7,8, 19]. This approach transformed the field of virology by enabling viruses to be isolated, quantified and experimentally manipulated. However, plaque-based detection inherently favours viruses capable of producing visible lysis under laboratory conditions. Thus, early virus discovery was strongly biassed towards lytic viruses infecting readily cultivable hosts, leaving large portions of viral diversity undiscovered.

The 1930s marked a shift inferring viral presence through host lysis to observations of virus structure with the arrival of electron microscopy. This method provided the first direct visualization of viral particles and enabled viruses to be classified based on morphological features, such as capsid organization, tail structures and the presence or absence of envelopes [20,22]. Morphological classification represented a major conceptual advance, allowing viruses to be grouped into structural families and forming the foundation for early viral taxonomy. Yet this framework, like plaque assays, was constrained by both technological and biological limitations, requiring cultivable viruses that could be propagated at high titres.

Advances in molecular biology further expanded the tools available for virus identification. Coinciding with these early molecular advancements, the International Committee on Taxonomy of Viruses (ICTV) published its first report in 1971, establishing the first initial viral species list. Simultaneously, the development of Sanger sequencing in the 1970s enabled the first direct sequencing of viral genes and genomes, providing a molecular framework to study viral diversity and evolution. However, early sequencing efforts remained relatively low throughput and typically required viral isolation prior to sequencing. The broader molecular revolution of the late twentieth century introduced new strategies for virus identification that no longer relied on particle visualization or host cultivation. In particular, approaches like PCR and Reverse Transcription-Polymerase Chain Reaction (RT-PCR) enabled sensitive and rapid detection of viral nucleic acids directly from biological samples, greatly expanding surveillance capabilities of known viruses without the need for viral cultivation [9]. However, these methods still depended on prior knowledge of viral sequences to design primers, restricting the detection to viruses closely related to previously characterized viruses.

While the use of PCR and RT-PCR expanded molecular surveillance for known viruses, it left the broader problem of viral diversity unaddressed. In order to partially overcome this limitation, researchers in the 1980s began targeting conserved viral genes within key viral groups as phylogenetic markers, which enabled the discovery of viruses carrying homologues of genes found in known viral lineages (see Fig. 1b and Table S1, available in the online Supplementary Material [10,23,38]). For example, in bacteriophages, conserved structural and replication-associated genes such as gp23, encoding the major capsid protein of T4-like phages [39,40], and the terminase large subunit (TerL) conserved broadly in tailed dsDNA phages, are used as a phylogenetic marker to infer similarity. Such markers enabled the classification of viruses into viral lineages. Similarly, in RNA viruses, RNA-dependent RNA polymerase (RdRp) was initially used as a genetic marker [41,42]. Despite the lack of a viral universal marker gene, these marker-gene approaches enabled broader surveys of viral diversity but were limited by their dependence on previously known conserved genes, restricting the detection of novel divergent viruses. This absence reflects both the unique viral diversity and evolutionary history but also highlights the limitations of marker-based classification.

Historical approaches have established the conceptual and methodological foundations of virus identification and classification. However, their limitations in requiring cultivation, conserved markers across species and limited detection on low-abundance viruses have motivated the development of current practices.

## Current and integrative approaches to virus identification

Current virus identification approaches have been fuelled by the transition from targeted, marker gene-based and low-throughput culturing and sequencing strategies to untargeted metagenomic and metatranscriptomic approaches, which capture DNA and RNA, respectively, capable of surveying entire viral communities. Advances in next-generation sequencing have made it possible to sequence all the nucleic acids within a community without the need for targeted amplification, freeing virus discovery from its historical dependence on known viral groups or predefined genetic markers. Instead, current virus discovery involves the recovery of viral sequences directly from complex mixtures of DNA and/or RNA derived from diverse biological communities. This conceptual and technical shift has led to the exponential rise in the discovery of novel viral species over the last decade. It has also introduced a computational hurdle. Specifically, using an untargeted approach makes extracting and identifying viral genomes from complex datasets difficult. To understand how the field of viromics is overcoming this limitation, we mapped the current state of virus discovery in the viral metagenomic field.

While the first virome was published in 2002 [13], the field did not expand substantially until the 2010s, when genome- and population-level analyses began to take off. For this review, we surveyed Google Scholar using the search terms (‘viral population’ OR ‘vOTU’) AND (‘viral metagenome’ OR ‘virome’) between January 2010 and March 2026, yielding an initial 2,083 results (see full list in Table S2). We excluded 1,580 studies comprising theses/dissertations, reviews, book chapters or those reporting a low number of viral operational taxonomic units (vOTUs). To ensure robust comparative analysis, the dataset was further restricted to studies reporting more than 1,000 de-replicated vOTUs, as well as targeted inclusions of less than <1,000 vOTUs from unique or underrepresented environments. This resulted in a total of 502 studies (summarized in Table 1) with the detailed metadata extracted from these final studies, focusing on assemblers, virus identification tools and vOTU counts, grouped into 3-year periods to highlight temporal trends.

**Table 1.** Summary of studies included in the database across time periods (2010–2026) The table presents the number of studies and predominant environments: host, marine, freshwater, soil, extreme and other environments (agricultural slurry, chaparral wildfire, plastisphere, food and public transit air). This table also shows the commonly used assemblers and virus identification tools for each period, along with the total number of de-replicated vOTUs and their mean±sd per study.

Publishing year	No. of studies	Environment	Main assembler	Main virus identification tool	Total no. of vOTUs	Mean and sd
2010–2013	13	Host (6), marine (5), freshwater (1), extreme (1)	CLC Genomics Workbench, Newbler, Velvet	blast	5,273	1,054.6±2,126.2
2014–2017	51	Host (34), marine (8), other (5), freshwater (2), extreme (2)	MIRA, Velvet, SPAdes, IDBA-UD	blast, HMMER, VirSorter	68,681	4,040.1±7,295.0
2018–2021	119	Host (56), other (23), marine (18), soil (16), freshwater (4), extreme (2)	MEGAHIT, SPAdes, Velvet	blast, VirSorter, VIBRANT, VirFinder, DeepVirFinder	31,843,750	403,085.4±33,288,800.4
2022–2025	303	Host (133), other (51), soil (45), marine (45), freshwater (20), extreme (9)	MEGAHIT, SPAdes, Trinity, Flye	VirSorter2, VIBRANT, blast, geNomad, DeepVirFinder	12,934,157	48,624.7±224,556.30.3
2026*	16	Host (9), marine (4), other (1), extreme (1), freshwater (1)	MEGAHIT, SPAdes, Flye	blast, PhaGCN, geNomad, VirSorter2, VIBRANT	744,132	49,608.8±97,671.80.8

*As of March 2026.

**Fig. 2.**
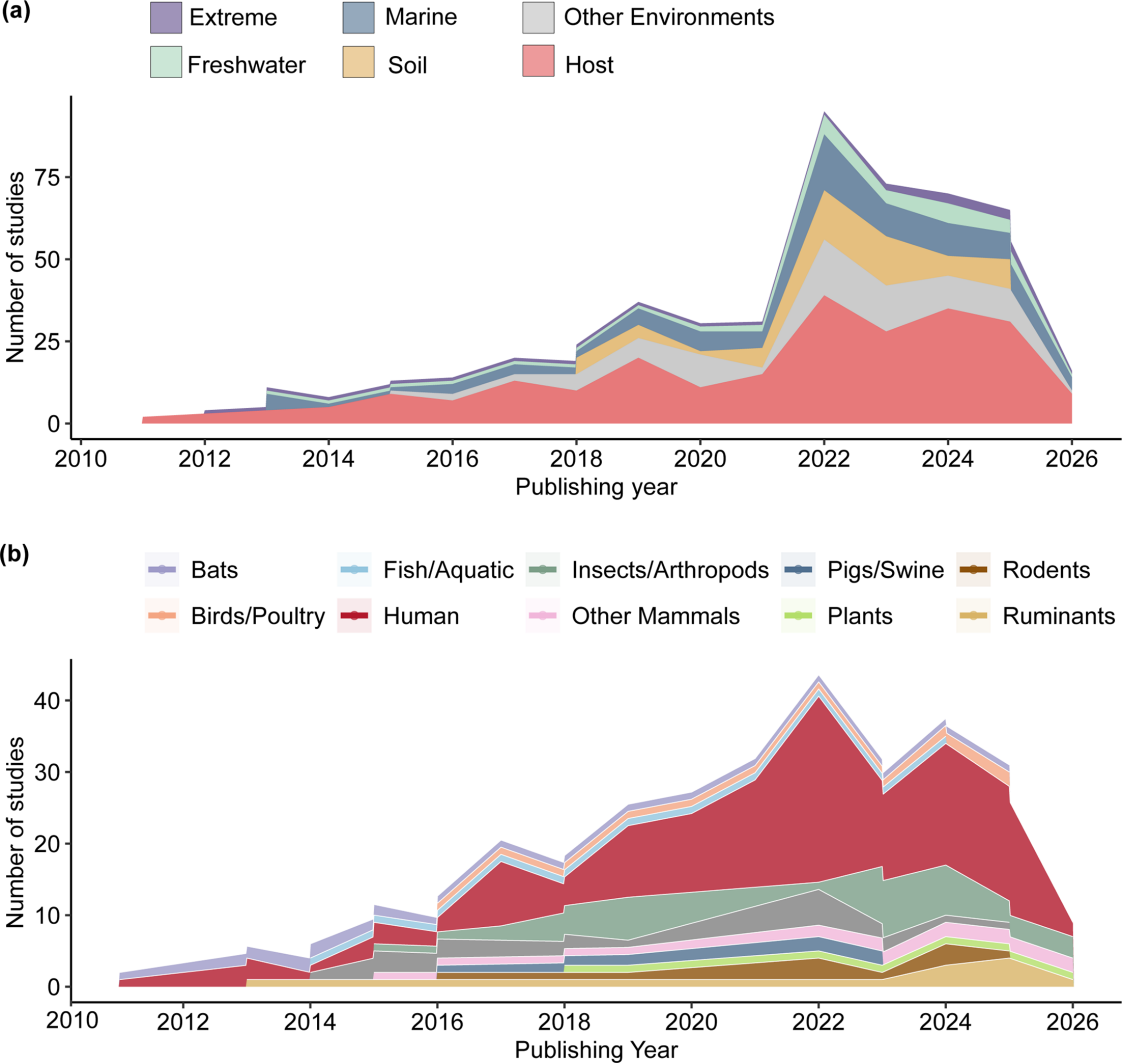
Temporal dynamics of viral detection studies across environments (2000–2026). (**a**) Density plot illustrating the temporal distribution of studies that sequenced within the six environment groups: extreme, freshwater, marine, soil, other and host (*n*=502 studies). (**b**) Stacked area plot showing diversification within the host environment group (2011–2026), with subcategories representing specific host types (*n*=238 studies). Area proportional to the number of studies published per year.

Early studies heavily focused on exploring aquatic environments [43,48] and simple clinical snapshots of human faecal or oral samples [49,51]. This initial emphasis on aquatic biomes was driven by early estimates, revealing high viral abundance [52]. Given that the marine environment does contain the overwhelming majority of the Earth’s viruses, researchers have focused their efforts on the oceans as a massive reservoir for discovering viral populations. This aquatic focus culminated in landmark global surveys including the J. Craig Venter Institute Global Ocean Survey [53], Malaspina [54] and the Tara Oceans global dataset [55,57], which the latter two helped establish foundational baselines for marine viral diversity, resulting in ~579,904 vOTUs in the most recent expansion of the Global Ocean Virome 2.0 dataset [58] (Fig. 2a).

Between 2014 and 2021, there were 170 out of the 502 total studies. Of those 170, 53.9% (*n*=90) was linked to human health, wildlife and livestock. This reflects a shift towards host-associated microbiomes and viromes (Fig. 2a, b). Aquatic environments (marine and freshwater) remained a core location for major viral metagenomic studies with 18.8% (*n*=32) being related to this area. This period of time also marked the emergence of research targeting complex soils with 9.4% (*n*=16) being dedicated to this area.

**Fig. 3.**
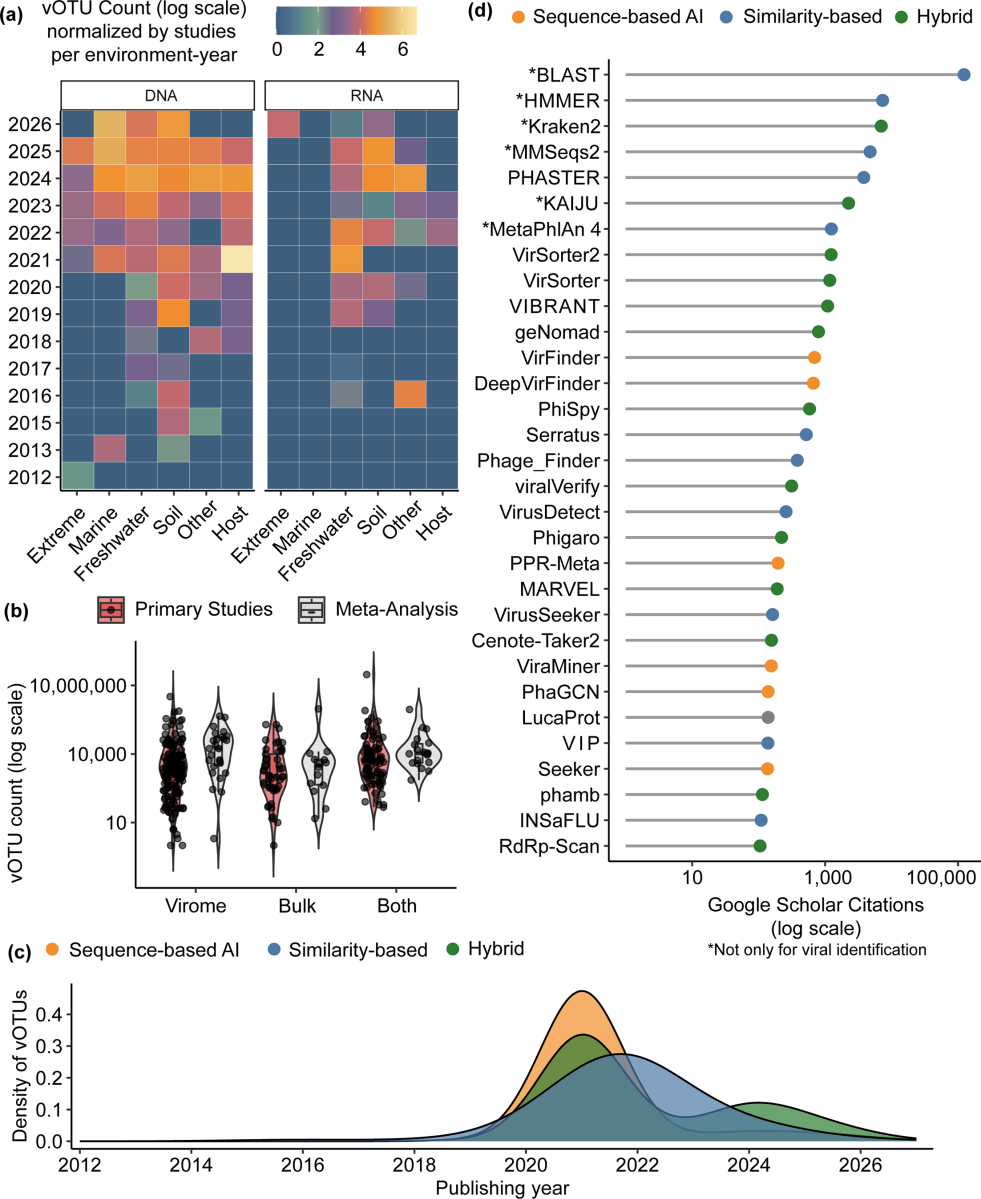
vOTU detection, dataset characteristics and tool usage in viral identification studies. (**a**) Heatmap showing the number of de-replicated vOTUs (log10 scale) reported per year (2012–2026), stratified by nucleic acid type (DNA and RNA) and environment (extreme, marine, freshwater, soil, other and host). We normalized the vOTU count based on the number of studies per environment per year to show whether viral discovery has become more efficient over time. (**b**) Distribution of vOTU counts (log10 scale) across study types (virome-enriched, bulk sequencing and both strategies), comparing primary studies and meta-analyses. Points represent individual studies; violin plots indicate data distribution. Differences among sequencing strategies are shown as descriptive patterns and were not subjected to formal statistical testing. (**c**) Density distribution of publication years weighted by the number of de-replicated vOTUs reported per study, stratified by viral identification methodology. Sequence-based AI methods correspond to machine learning or deep learning approaches, similarity-based methods correspond to homology or reference-dependent approaches and hybrid methods include studies combining tools from multiple methodological categories. Density values were calculated using raw vOTU counts without log transformation. This panel illustrates temporal shifts in viral identification strategies and their association with increasing vOTU discovery over time. (**d**) Most frequently cited viral detection tools across studies, shown on a log10 scale. Points represent citation frequency, coloured by methodological category (sequence-based AI, similarity-based and hybrid). Citation counts were retrieved from Google Scholar in April 2026, based on the original publication describing each tool. The tools marked with an asterisk (*) are not just for viral detection.

The current landscape (2022–2026) has seen an explosive surge in data generation for viral metagenomics, with our literature survey capturing 319 out of the 502 metagenomic studies and databases published during this short timeframe (see Table 1). This era is characterized by the consolidation of sequences into massive databases such as Gut Virome Database [59], Gut Phage Database [60], Metagenomic Gut Virus Catalogue [61], ViromeDB [62], Global Soil Virus (GSV) Atlas [63], Groundwater Virome Catalogue (GWVC) [64], Global Deep-sea Sediment RNA Virome 2.0 (GDSR2.0) [65], Chinese Gut Viral Catalogue (cnGVC) [66] and MetaVirus Resource (MetaVir) [67]. The construction of these large viral databases highlights the rapid increase in efficiency of viral detection from 2000 to 2026 (Fig. 3a). This also coincided with a targeted push into extreme and atypical environments, extending viral sampling into habitats as diverse as hydrothermal vents, wildfires, the plastisphere and the cheese virome (Fig. 2a).

As viral ecology has expanded into more unique and diverse environments, the associated bioinformatic pipelines used for virus identification have had to account for several biases introduced during wet lab experiments. One of the most significant biases in early viral metagenomics data arises from the need to amplify low quantities of DNA. In environments where viral biomass is extremely low, researchers frequently rely on multiple displacement amplification (MDA) to generate enough material for sequencing. However, the Phi29 polymerase used in the MDA process exhibits a significant amplification bias towards small ssDNA [68]. Therefore, in studies within our dataset, where MDA was utilized, the abundance of ssDNA viral families will most likely be higher. Interestingly, our analysis reveals that MDA usage was drastically higher in the field’s early years, with 46% of the 13 studies utilizing MDA between 2010 and 2013, compared to only 5% of the 319 studies between 2022 and 2026. This steep decline coincides with the advent of better library preparation, extraction and sequencing methods available as well as rigorous method testing [69].

Traditional ‘bulk’ metagenome sequencing, which captures the entire microbial community, enables the identification of free viruses but also integrated prophages and their bacterial hosts. It also captures signals of actively infecting viruses, which often comprise the majority of recovered viral sequences [70]. The primary limitation of this approach is the overwhelming ‘noise’; because host cellular genomes are larger than viral genomes, the viral signal is often drowned out unless the sequencing depth is exceptionally high.

To circumvent this, researchers often aim to enrich the virome by isolating viral-like particles. This is typically achieved through size fractionation using a 0.22 µm filter or chemical flocculation–precipitation, followed by density gradient separation to isolate virions before extraction [71]. Alternatively, the extraction process itself can enrich viruses by utilizing commercial kits designed with specialized lysis buffers and binding matrices optimized specifically for viral nucleic acids [72]. Our data analysis underscores the advantage of a dual strategy, with those studies utilizing a combination of both methods recovering a higher mean number of vOTUs compared to those relying solely on bulk metagenome or virome approaches (Fig. 3b). Overall, the extraction and sequencing strategies utilized across these studies are only a small part of the current virus discovery pipeline. Genomic analysis alone is not suitable to answer the whole spectrum of questions that researchers come up with and might not be ideal in every situation [73]. Virus isolation, culture-based assays and serology still play a very important role in epidemiological studies and defining host range [74,75]; electron microscopy is still widely used to understand how viruses are organized at the molecular level and how they behave in different tissues and conditions, including infections [76,78]; experimental validation is still the most trustworthy line of evidence, which can confirm or disconfirm bioinformatic predictions, which could return false positives due to the presence of fragmented or residual viral nucleic acids [79]. Because of that, all these methods are still actively used in viral identification and characterization, and it is often necessary to integrate more than one of them together to complement the evidence they offer and put together an explanation that is biologically relevant [80].

Today, the success of large-scale viromics studies relies on computational power driven by assembly and virus identification tools. Within this framework, virus identification is now largely driven by assembled sequencing data. Raw short reads are computationally assembled into longer contigs, after which viral sequences must be distinguished against a background dominated by cellular and other non-viral genetic material. As a result, virus identification has become a computational challenge, one that demands the integration of multiple lines of evidence to accurately classify and interpret the recovered genomic fragments. The pairing of specific assemblers with virus identification tools reflects methodological compatibility as well as the field’s evolving preferences, often shaped by dataset characteristics, sequencing technologies and the desired resolution of viral recovery.

Analysis of assembler usage across 502 studies (Tables 1 and S2) revealed clear trends in their usage over time and impact on vOTU detection. From 2010 to 2017, most studies reference assemblers like Newbler [81], Velvet [82] and CLC Genomics Workbench (QIAGEN digitalinsights.qiagen.com), which were originally designed for isolated genomes and low-complexity datasets. As metagenomics grew more widespread, the field shifted towards tools better suited for complex microbial communities and highly variable sequencing depths, such as SPAdes [83] and Megahit [84]. This transition matches with a noticeable increase in the number and continuity of recovered viral contigs, ultimately enhancing vOTU detection over time (Fig. 1b). It is important to mention the transition from general assemblers to metagenomics-specific assemblers, including MetaSPAdes [85], MetaViralSPAdes [86], MetaVelvet [87] and MetaFlye for long-read sequencing [88]. These specialized implementations incorporate algorithmic adjustments tailored to metagenomic complexity. The choice of assembly tool directly impacts vOTU detection, since different assemblers yield substantially different numbers of viral contigs and affect both the length and completeness of recovered viral genomes, as well as the inferred viral community composition. Therefore, assembler choice is not a neutral technical detail but a key methodological decision that shapes which vOTUs are detected, how well they are resolved and ultimately how viral diversity and community structure are interpreted across studies [89].

Following assembly, the subsequent step in vOTU detection involves virus identification tools, which can be divided into four main groups based on their methodological approach: (i) similarity-based, (ii) sequence-based AI, (iii) structure-aware AI and (iv) hybrid that combine two or more of these approaches, with structure-aware AI methods representing a more recent development with only one reported tool (Tables 2 and S3). Similarity-based approaches rely on reference databases to detect and classify viral sequences through both sequence-level comparisons and gene content–based profiles. In contrast, sequence-based AI approaches extract numerical features from sequences and apply machine or deep learning (DL) models to recognize viral genome patterns, including those from novel or highly divergent viruses lacking close homologues. Structure-aware AI methods predict protein structures to inform virus identification and functional annotation. Hybrid approaches integrate multiple elements, often combining sequence similarity, gene content, numeric features and/or structural information to improve detection sensitivity and classification accuracy.

**Table 2.** Summary of virus identification tools grouped by methodological approach The table lists the number of tools in each category (similarity-based, sequence-based AI, structure-aware AI and hybrid) and the viral targets they are designed to detect (all viruses, DNA and RNA viruses). For each group, the table indicates whether the tool provides taxonomic classification and/or functional annotation of detected viral sequences.

Approach	No. of tools	Viral target			Taxonomy (yes/no)	Functional annotation (yes/no)
		All viruses	DNA virus	RNA virus		
Similarity-based	36	23	8	5	Yes: 29 No: 7	Yes: 23 No: 13
Sequence-based AI	28	14	12	2	Yes: 6 No: 22	Yes: 5 No: 23
Structure-aware AI	1	0	0	1	Yes: 0 No: 1	Yes: 1 No: 0
Hybrid	31	14	13	4	Yes: 18 No: 13	Yes: 17 No: 14

The most commonly used virus identification tools described in Table 1 are blast [90], VirSorter2 [91], VIBRANT [92], geNomad [93] and DeepVirFinder [94]. Based on the aforementioned subdivision by methodological approach, blast is classified as a similarity-based tool, DeepVirFinder as a sequence-based AI tool and VirSorter2, VIBRANT and geNomad as hybrid tools (Table S3). Throughout this review, we examine in detail the methods employed by these prominent tools, highlighting their respective strengths and limitations in accurately characterizing viral sequences from complex metagenomic datasets.

To systematically evaluate these approaches, we compiled a list of 95 publicly available virus identification tools capable of detecting viral sequences from unclassified metagenomic data (Tables 2 and S3). For each tool, we provide information on its primary methodological approach as well as the viral groups it targets: (i) all viruses, (ii) DNA viruses or (iii) RNA viruses. This classification allowed us to cross-reference tool usage across the literature and evaluate how different approaches contribute to vOTU detection over the years. In this review, we discuss each methodological approach, highlighting their principles, strengths, limitations and impact on viral detection in metagenomic studies.

### Sequence- and gene content–based virus identification

All similarity-based approaches compare query nucleotide or protein sequences directly to reference databases using alignment methods that match positions across sequences [95]. A sequence alignment represents a hypothesis of shared evolutionary history, assuming that nucleotides or amino acids at aligned positions have diverged from common ancestors [95,96]. These methods can be subdivided into sequence-based and gene content–based virus identification tools. Sequence-based methods directly analyse the nucleotide or amino acid composition of raw contigs using local alignment tools like blast [90], MMseqs [97] and DIAMOND [98,99], which perform exact or near-exact matches against curated repositories such as those maintained by the National Center for Biotechnology Information (NCBI) and the European Bioinformatics Institute (EBI). Local alignment tools aim at identifying conserved or homologous regions, enabling similarity analysis, functional inference and taxonomic assignment. In contrast, gene content–based methods, such as HMMER [96], employ local alignments to detect similarities between query amino acid sequences and hidden Markov model (HMM) profiles. These profiles are built from global alignments of proteins deposited in large HMM databases like Pfam (now integrated into Interpro) [100] and/or viral specific HMM databases such as vFams [101], eFams [102], VOGdb [103], PHROG [104] and The RNA Viruses in Metatranscriptomes (RVMT) [105], offering high functional specificity or longer contigs but potentially missing viruses lacking close homologues and annotated ORFs [101].

The survey of 502 studies reveals preferences for certain bioinformatic tools (Table 1), particularly similarity-based approaches such as blast and HMMER, hybrid approaches like geNomad and VirSorter2 and sequence-based AI approaches such as DeepVirFinder. Fig. 3(c) illustrates this trend by showing the density of de-replicated vOTUs identified from 2010 to March 2026 using similarity-based, sequence-based AI and hybrid tools. This figure illustrates temporal shifts in viral identification methodologies and their association with increases in vOTU discovery over time. Similarity-based tools account for the majority of vOTUs across this period, with a marked increase corresponding to the higher number of studies conducted between 2022 and 2025 (*n*=303). In the same period, vOTUs identified by sequence-based AI and hybrid tools show a noticeable rise, with a prominent peak of vOTUs identified by sequence-based AI tools around 2023. This shift likely reflects their ability to overcome limitations of similarity-based approaches and to integrate strengths from multiple methodologies.

Although similarity-based approaches have identified the majority of vOTUs across the surveyed studies and garnered the most citations in Fig. 3(d), their effectiveness remains intrinsically tied to the genetic diversity and quality of sequences deposited in reference databases as well as the diversity present within the samples themselves. These databases are subject to biases that affect the representation of viral diversity. It is estimated that only a small fraction of the viral diversity found in prokaryotic hosts is represented in databases, due to limitations associated with the isolation and assembly of viral genomes [15]. Consequently, many vOTUs are represented by few genomes, which are often incomplete or low quality. In the absence of close homologues, a large portion of viral genomic sequences is labelled as unknown, also referred to as ‘viral dark matter’. These unknown sequences are frequently discarded and do not undergo subsequent bioinformatic analyses following alignment against a reference database [106]. Beyond database limitations, the similarity-based approaches assume collinearity (linearly conserved nucleotides), which fails for viruses with high mutations, recombination, horizontal gene transfer and gene duplications [107,108].

### Sequence-based AI virus identification

To assess the limitations inherent in similarity-based viral detection, recent studies have increasingly used sequence-based AI approaches, either standalone or hybrid with similarity-based methods. Sequence-based AI approaches leverage machine learning (ML) and DL techniques to learn viral genome patterns from reference databases and identify viruses in unclassified contigs as short as 500 bp [108,109]. In contrast to similarity-based approaches, sequence-based AI approaches rely on alignment-free numerical features derived from sequences. By assessing sequence features without the need for alignment to reference genomes, these tools are able to detect conserved genomic signatures in divergent or novel viruses that are often overlooked by similarity-based approaches. This provides robustness to fragmented contigs, low-complexity regions and recombination events, ultimately enhancing sensitivity and uncovering previously uncharacterized viral diversity [94,108, 110].

Sequence-based AI virus identification tools have been published since 2017 [109] with publication rates accelerating between 2019 and 2022 [94,110,112] according to our survey (Table S3). This surge corresponds to the rise in metagenomic studies and the development of more complex algorithms over time [108]. The earliest sequence-based AI tools relied on relatively simple ML models, such as random forest and logistic regression that were trained on computationally simple alignment-free sequence-derived features including k-mer frequencies, codon usage bias and GC content. These features are effective for viral detection since viral genomes often exhibit distinct compositional patterns compared to their host or other microbial sequences, reflecting varying nucleotide usage, coding strategies and genome organization [92,109]. Early implementations primarily focused on simple classification tasks, such as binary classification of viral and non-viral contigs. The development of these tools represented a paradigm shift in virus identification; these models laid the foundation for more sophisticated algorithms, enabling the application of advanced methods from other fields to the challenges of metagenomic virus discovery.

The most highly cited sequence-based AI tools on Google Scholar as of March 2026 are VirFinder [109], DeepVirFinder [94], PPR-Meta [112], ViraMiner [110] and PhaGCN [111], while the leading hybrid tools are VIBRANT [92], VirSorter2 [91] and geNomad [93] (Fig. 3d). This pattern aligns with the compilation of studies outlined in Table 1. In most of these studies, these sequence-based AI tools were used either exclusively or in combination with similarity-based tools within multi-tool pipelines. To further illustrate the diversity of sequence-based AI virus identification approaches, these tools can be divided into ML and DL techniques, which differ in the way they learn (numeric features) and interpret viral sequence features (models used).

The most cited ML virus identification tools VirFinder [109], VIBRANT [92] and VirSorter2 [91] present notable differences in both feature selection and model architecture that influence their performance and overall applicability. For instance, VirFinder is an interpretable and fast tool that uses k-mer-based features and a logistic regression model to classify short contigs as viral or non-viral. However, this tool tends to underperform compared to both VirSorter2 and VIBRANT by producing more false positives, which makes its predictions less reliable than those of VirSorter2 and VIBRANT [113]. In contrast, hybrid tools such as VIBRANT and VirSorter2 combine similarity-based information with ML classifications to improve the detection of fragmented or divergent viral sequences. Specifically, VIBRANT employs a neural network with a ‘v-score’ metric to estimate the probability that a contig contains virus-like protein annotation signatures, thereby enabling viral detection [92]. In contrast, VirSorter2 applies multiclassifier random forest models based on hallmark protein annotations [91].

VirSorter2 features multi-classifier and broad-spectrum viral detection, moving away from a single cohesive model towards a modular framework [91]. Contigs first undergo functional annotation and HMM-based hallmark gene detection, after which VirSorter2 feeds a fixed set of 27 genomic features extracted from the annotated contigs directly into its five group-specific random forest classifiers. This architecture uses five specialized classifiers to detect diverse viral groups: dsDNA phages, ssDNA viruses, RNA viruses, large Nucleocytoviricota and underrepresented groups. Its modularity enables updating individual classifiers or adding new models to classify viral groups as they are characterized without overhauling the entire algorithm [91]. Benchmarking has shown that VirSorter2 [91] outperformed VIBRANT on seawater and gut biomes, while VIBRANT outperformed VirSorter2 on soil biome [113,114]. The main limitations of VirSorter2 are related to reduced sensitivity on short contigs (<3 kb) and non-Caudovirales due to limited hallmark genes [91,113, 114]. Additionally, according to recent benchmarking studies, VirSorter2 presents high false positives on eukaryotic sequences, heavy computational demands and decreased accuracy. This further highlights the need for thorough output analysis and potential combination with other tools to reduce false positives. Notably, of the 107 studies that used VirSorter2 listed in Table 1, 81.3% (*n*=87) combined VirSorter2 with other virus identification tools, including similarity-based approaches such as HMMER and blast, sequence-based AI approaches such as DeepVirFinder and PHAGCN, and hybrid approaches such as VIBRANT and geNomad.

DL tools are able to complement ML tools by improving the detection of novel or highly divergent viruses that ML tools might miss [94]. ML classifiers, like the random forest in VirSorter2, depend on predefined features, while DL classifiers automatically learn features directly from the input contigs. ML excels with viruses similar to its training data but struggles with distant viruses, novel patterns or subtle signals beyond those features. In contrast, DL uncovers hierarchical, non-linear patterns, enabling stronger generalization to unknown viruses without manual feature selection [94,108, 110]

Among the most cited DL tools (Fig. 3d), DeepVirFinder uses convolutional neural networks (CNNs), which automatically learn patterns in short DNA subsequences by scanning sequences with small filters that capture local motifs, analogous to how the visual system detects edges and shapes in images [94]. These CNNs are trained on k-mer-encoded sequences to distinguish viral from non-viral contigs, demonstrating higher sensitivity than traditional ML tools at shorter contig lengths [108]. ViraMiner extends this approach with dual-branch CNNs that integrate nucleotide input and k-mers, outperforming DeepVirFinder on human microbiomes [110]. PhaGCN employs a graph convolutional network framework that leverages sequence context and taxonomic relationships for superior taxonomic assignment over conventional classifiers [111].

In contrast with these tools, geNomad [93], one of the most robust virus identification tools, identifies viruses and plasmids through a hybrid pipeline combining a deep neural network, which learns complex patterns in raw nucleotide sequences across multiple processing layers (sequence branch) with marker gene-based classification (marker branch) being considered in this review as a hybrid tool. Specifically, geNomad markers were defined by building a comprehensive database of protein profiles from viruses and plasmids, with alignments retrieved from verified and highly curated sources, including VOGdb [103], PHROG [104] and RVMT [105], to identify the profiles that are informative for sequence classification. These markers were then assigned to ICTV taxa using alignment and a majority vote function. This approach allows the programme to provide a reliable and relatively robust taxonomic classification (which can go up to the species level in the latest version of the programme) for the putative viral sequences identified. Other than markers and sequence-based approaches, geNomad is also equipped with a DL module consisting of a gene-based and a sequence-based classifiers, of which the respective outputs are aggregated by a neural network which is able to weight the contribution of the models, returning a final score.

Despite advances in genome-based virus identification, all current approaches remain constrained by their reliance on sequence similarity and protein family homology. Even hybrid frameworks that integrate gene content, sequence composition and ML continue to depend, either directly or indirectly, on patterns derived from known viral genomes. As a result, highly divergent viruses that lack detectable sequence or protein cluster homology remain difficult to identify, contributing to the persistence of viral ‘dark matter’ [15,106, 108].

## The future of virus identification

**Fig. 4.**
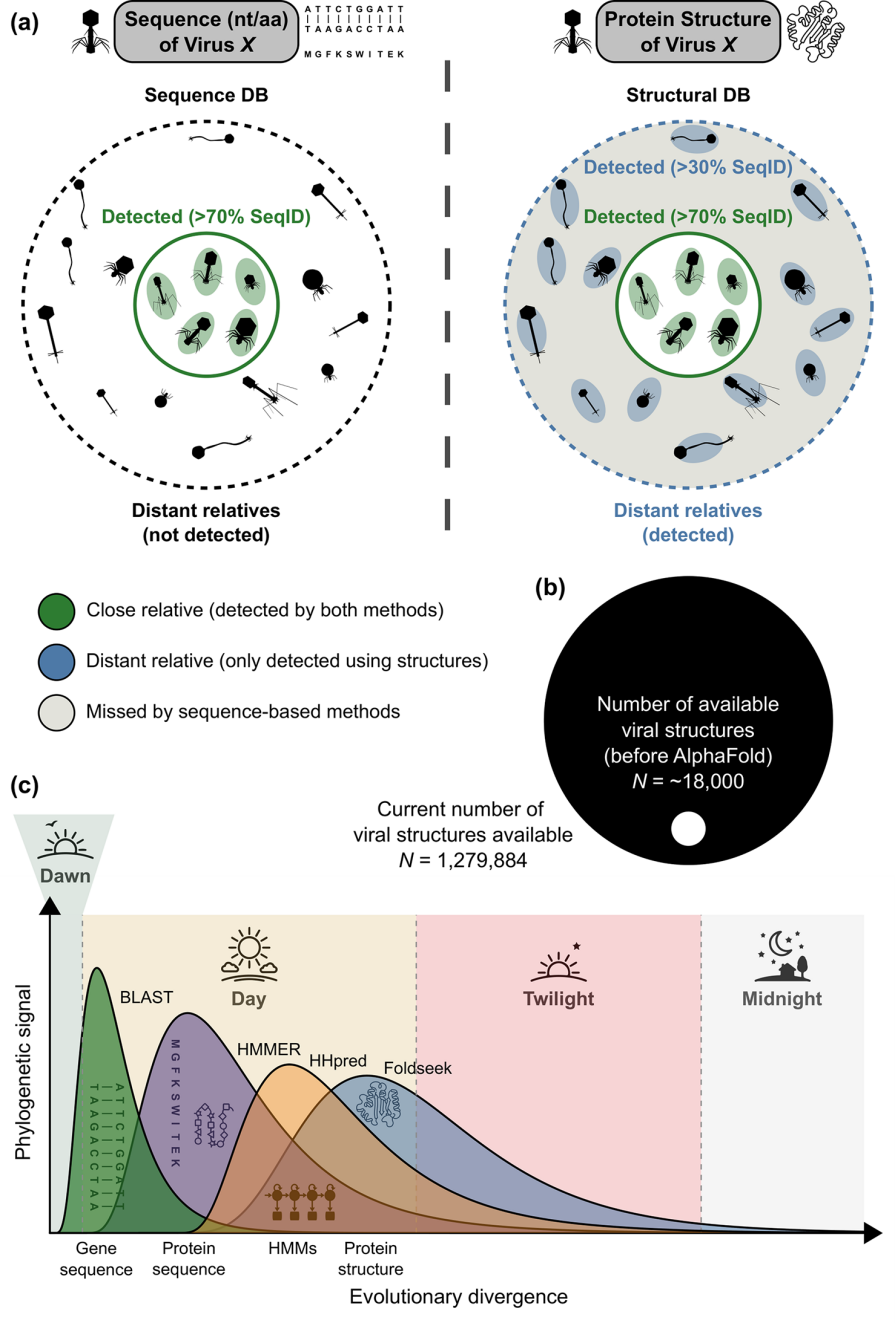
Virus identification is enhanced through protein structural comparison. (**a**) The use of structural databases enables the identification of viruses at lower sequence identity thresholds, increasing the likelihood of detecting viral sequences across greater evolutionary distances. (**b**) Plot showing the increase in the number of publicly available viral protein structures following the release of structural databases based on AlphaFold predictions. (**c**) Different representations of predicted ORFs, ranging from the nucleotide sequence to translated protein sequence, HMM profile and predicted protein structure, capture progressively deeper levels of virus signal, each suited to distinct scales of virus identification and phylogenetic reconstruction (adapted from [139].

One emerging direction to address these limitations is the incorporation of protein structure as a complementary layer of information for virus discovery. Unlike primary sequence, protein structures are often conserved across deep evolutionary timescale even among proteins that share little to no detectable sequence similarity [115] (Fig. 4a). This property is particularly relevant for viruses, where structural features, such as capsid and nucleocapsid folds, can remain conserved despite extensive sequence divergence [116,117].

Recent advances in AI-driven protein structure prediction, most notably AlphaFold [118], combined with decreasing computational costs, have made it feasible to incorporate structure-based information into large-scale virus discovery workflows. In parallel, rapid structural comparison tools such as DALI [119], Foldseek [120] and Reseek [121] enable efficient searches across vast protein structure space. Together, these developments suggest that virus identification can extend beyond sequence similarity into structural homology, providing a means to detect viruses that are invisible to traditional sequence-based approaches.

Early applications of this idea have shown that viral proteins lacking detectable sequence homology can still be annotated through structural similarity searches, recovering functional and evolutionary signals that are otherwise missed [122]. Pipelines such as Phold have formalized this concept by incorporating structure-aware homology searches into phage genome annotation [123]

In parallel, structural information is increasingly being integrated directly into AI models for virus identification. For RNA viruses in particular, the conserved RdRp has served as a key entry point for detecting highly divergent lineages. While RdRp has long been used as a phylogenetic marker, traditional approaches based on sequence similarity alone can fail when divergence is high. Newer DL models address this limitation by incorporating structural representations alongside sequence features, enabling the detection of RdRp proteins that fall below conventional similarity thresholds. Recent large-scale applications of these approaches, such as the LucaProt method, a DL algorithm which integrates sequence and structural features of a large and highly curated set of viral RdRp proteins (*n*=5,979), as well as a negative one of non-viral proteins (*n*=229,434), have substantially expanded the known RNA virosphere, identifying hundreds of thousands of putative viral sequences and uncovering previously unrecognized diversity across global ecosystems [124].

Taken together, these developments point to a broader conceptual shift in virus identification. Rather than relying on a single source of signal, future approaches are likely to integrate multiple layers of information, including sequence, structure and learnt representations derived from deep neural networks. Structure-aware models are particularly promising for resolving viral dark matter, as they capture conserved functional and evolutionary relationships that are not apparent at the sequence level.

The recent development of large-scale viral structural databases now supports this shift. These databases include the Big Fantastic Virus Database (BFVD) [125], Viro3D [126], the Nomburg’s virome database at ModelArchive [127], Viral AlphaFold Database (VAD) [128] and Meta-virus resource (MetaVR) [129]. Taken together, these resources offer access to 1,279,884 folded viral protein sequences (Fig. 4b). This is a staggering increase, considering that the total number of protein structures available before AlphaFold came out was ~180,000 [130], with less than 10% (~18,000) being viral proteins [126,131] (Fig. 4b).

Most of these databases use AlphaFold2 as a predictive model, which compares well with its newer versions (e.g. AlphaFold3) [132], while offering greater reliability and stability in terms of predictions [133]. This model is currently required by the AlphaFold DB community for uploading contributions to the database (European Bioinformatics Institute 2026). Even though the AlphaFold (either 2 or 3) is currently the most used framework for compiling databases of predicted viral protein structures, other methods showing comparable results are available, such as RoseTTAFold [134], ESMFold [135], Boltz-2 [136], Chai-1 (The Chai discovery team 2024), HelixFold3 [137], Protenix (The ByteDance AML AI4Science Team 2025), OpenFold3 (The OpenFold3 Team 2025) and IntelliFold-2 [138]. This scenario is changing rapidly, and comprehensive benchmarks for these tools on viral proteins are still lacking, so the preference for AlphaFold may change in the future.

This abundance of methods and databases is catalysing a new era of virus identification and classification. New structural phylogenetic methods are emerging [139] to capture the phylogenetic signal that lies in protein structures [140], which will likely have an impact on the way we identify and classify viruses, shedding light on deeper evolutionary relationships that cannot be accessed by relying on nucleotide or amino acid sequence alone, as well as on HMMs (Fig. 4c). A more robust and deeply resolved phylogenetic framework grounded in structural information could directly help define reliable markers for virus identification. Unlike sequence-based markers, which are prone to saturation and homoplasy over long evolutionary distances [141], structurally defined markers would retain phylogenetic informativeness even across highly divergent lineages [142], making them particularly suitable for the identification of viruses that fall outside the known sequence space.

One of the most promising approaches in this field consists of making protein structures easily alignable and therefore usable for efficiently getting matrices that can be employed for inferring phylogenies, a capacity with direct implications for virus identification, since viruses that have diverged beyond the point of detectable sequence similarity may still share conserved structural folds. This is done by using structural alphabets, which consist of converting the 3D structure of a protein into a sequence of characters that represent a simplified version of the original protein structure [143]. Foldseek [120] implements one of the most popular structural alphabets so far, using a set of 20 characters called three-dimensional interaction characters (3Di characters) to represent the tertiary interactions of each amino acid with its spatially closest residue in the space. Another method based on a similar approach is Reseek, with the difference of using a much larger alphabet (a ‘mega-alphabet’ of ∼10^11^ letters) for representing the structure as a sequence, which gives to the method the capacity of capturing more subtle relationships between structures, improving sensitivity to remote homologs [121], a key property when trying to assign taxonomic identity to highly divergent or novel viruses. Other alphabets for the detection of remote homologues, such as TEA [144], are under development and have yet to be tested extensively.

On the other hand, Foldseek’s 3Di characters have already been used to develop methods to efficiently get multiple sequence alignments of protein structures, such as FoldMason [145], as well as to identify structural core genes, like Unicore [146], which uses the ProstT5 [147] protein language model to directly obtain sequences of 3Di characters from amino acid sequences, facilitating and speeding up large-scale phylogenetic analysis with protein structural information. The ability to define and align structural core genes is particularly valuable for virus identification, as it enables the construction of robust reference phylogenies against which unclassified viral sequences, including those recovered from metagenomic datasets, can be placed, even when only predicted structural information is available.

Another method which builds on the same 3Di alphabet is Foldtree [142], which uses matrices obtained from comparing sequences of 3Di characters to reconstruct distance-based trees. These characters can be used alone or in combination with amino acids under neighbour joining or maximum likelihood frameworks [142,148], and the best combination for different cases, as well as the utility and performance of the 3Di approach itself [149], is still under debate [139,148]. Furthermore, combining sequence and structural information has also been shown as a possible way to improve the reliability of support values, such as the multistrap approach for bootstrapping [150], an important consideration when attempting to confidently assign novel viruses to known clades. Despite these advances, substitution matrices and evolutionary models specifically designed for 3Di characters remain underdeveloped, with only a limited number currently available [120,151], which may limit the accuracy of phylogenetic placements for highly divergent viral lineages.

Building on these methodological developments, recent efforts have also extended beyond distance-based and maximum likelihood approaches to incorporate structural information within a Bayesian framework, with new packages like FoldBeast already available [152]. For virus identification, Bayesian methods, which combine a prior probability with a likelihood (derived from data and evolutionary models) to compute the posterior probability of hypotheses [153], are especially relevant as they allow the integration of prior knowledge about viral diversity and can provide probabilistic estimates of clade membership for uncharacterized viruses. However, the application of clock models to structural data is still in its early stages, and further studies are required to determine the extent to which structural information captures a reliable temporal signal [139,142], a question that bears directly on whether structural phylogenetics can eventually support not just classification but also the inference of viral emergence timescales and evolutionary origins.

An example of the power of these new structural approaches in virology is well represented by the recent study by Mifsud *et al*. [154], in which the evolutionary history of the *Flaviviridae* family was reconstructed with unprecedented detail, with protein structure predictions employed to define the relationships among distant groups, define major clades, discover new members and define novel and acquired proteins across different genera. Importantly, this kind of structural phylogenetic resolution, reaching evolutionary relationships invisible to sequence-based methods, directly informs virus identification, providing a framework within which highly divergent or novel viruses can be placed and recognized even in the absence of meaningful sequence similarity. Moreover, the integration of structural phylogenetics into viral classification schemes could contribute to a more universal and evolutionarily coherent taxonomy, in which the boundaries of viral families and higher-order groupings are defined not by arbitrary sequence similarity thresholds, but by genuine shared evolutionary history reflected in protein architecture.

Looking forward, the next generation of virus discovery tools will likely combine structure-based homology, DL and large-scale metagenomic data (including further expanded protein structure databases) within unified frameworks. Equally important will be the incorporation of experimental validation to confirm computational predictions, together with ecological and environmental context to better understand viral distributions and functions. This integration would greatly enhance our ability to detect and classify viruses across many different scenarios, from low-diversity population-based studies in which closely related strains need to be tracked to high-divergence phylogenomic analyses of distantly related taxonomic groups. Such approaches have the potential not only to improve detection of highly divergent viruses but also to enhance functional annotation, evolutionary inference and ecological interpretation. As these methods mature, they may substantially reshape our view of the virosphere and provide a more complete understanding of viral diversity across ecosystems.

## Supplementary material

10.1099/mgen.0.001785 Table S1.

10.1099/mgen.0.001785 Table S2.

10.1099/mgen.0.001785 Table S3.

## Abbreviations

AI: artificial intelligence
CNN: convolutional neural network
3Di characters: three-dimensional interaction characters
DL: deep learning
HMM: hidden Markov model
ICTV: International Committee on Taxonomy of Viruses
MDA: multiple displacement amplification
ML: machine learning
RdRp: RNA-dependent RNA polymerase
vOTU: viral operational taxonomic unit
